# Dryocrassin ABBA, a novel active substance for use against amantadine-resistant H5N1 avian influenza virus

**DOI:** 10.3389/fmicb.2015.00592

**Published:** 2015-06-16

**Authors:** Changbo Ou, Qiang Zhang, Juan Wang, Guojiang Wu, Ningning Shi, Cheng He, Zengping Gao

**Affiliations:** ^1^College of Animal Science, Henan Institute of Science and TechnologyXinxiang, China; ^2^Key Lab of Animal Epidemiology and Zoonosis, Ministry of Agriculture, College of Veterinary Medicine, China Agricultural UniversityBeijing, China; ^3^School of Chinese Materia Medica, Beijing University of Chinese MedicineBeijing, China; ^4^College of Life Science, Agricultural University of HebeiBaoding, China; ^5^College of Pharmacy, Hebei Medical UniversityShijiazhuang, China

**Keywords:** dryocrassin ABBA, influenza virus H5N1, phloroglucinol compound, antiviral activity, *Rhizoma Dryopteridis Crassirhizomatis*, amantadine

## Abstract

The occurrence of multi-drug resistant highly pathogenic avian influenza virus (HPAIV) strains highlights the urgent need for strategies for the prevention and control of avian influenza virus. The aim of our current study is to evaluate the antiviral activity of dryocrassin ABBA isolated from *Rhizoma Dryopteridis Crassirhizomatis* (RDC) against an amantadine-resistant H5N1 (A/Chicken/Hebei/706/2005) strain in a mouse model. Post inoculation with HPAIV H5N1 virus in mice, the survival rate was 87, 80, and 60% respectively in the 33, 18, and 12.5 mg/kg dryocrassin ABBA-treated groups. On the other hand, the survival rate was 53 and 20%, respectively in the amantadine-treated group and untreated group. Mice administered with dryocrassin ABBA or amantadine showed a significant weight increase compared to the untreated group. Moreover, 33 and 18 mg/kg dryocrassin ABBA have decreased lung index (*P >*0.05) and virus loads (*P <*0.01) compared to the untreated group on day 7. Also, on day 7 bronchoalveolar lavage fluid pro-inflammatory cytokines (IL-6, TNF-α, and IFN-γ) decreased significantly (*P <*0.01) while anti-inflammatory cytokines (IL-10 and MCP-1) were increased significantly (*P <*0.01) in the 33 and 18 mg/kg dryocrassin ABBA-treated groups compared to the amantadine group and the untreated group. Moreover, the concentrations of IL-12 in drug-treated groups were significantly (*P <* 0.01) lowered compared with the untreated group. Based on the above we conclude that orally administered dryocrassin ABBA provided mice protection against avian influenza virus H5N1 by inhibiting inflammation and reducing virus loads. Dryocrassin ABBA is a potential novel lead compound which had antiviral effects on amantadine-resistant avian influenza virus H5N1 infection.

## Introduction

Highly pathogenic avian influenza virus subtype H5N1 (HPAIV H5N1) is responsible for large economic losses in the poultry industry and poses a serious threat to public health (Kaye and Pringle, 2005). The first step in decreasing the risk of human infection is to control the prevalence of HPAIV in domestic poultry (Peiris et al., 2007). Enhanced bio-security measures, such as surveillance, restriction of poultry movement and eradication of infection foci are basic principles for the control of HPAIV H5N1 epidemics in poultry, but these have not prevented the spread of the virus since 1997 (Capua and Alexander, 2006; Sarachai et al., 2014). Recently, several types of avian influenza virus vaccines have been introduced in some developing countries as a major control tool to reduce the overwhelming socioeconomic impact of HPAI H5N1 outbreaks in poultry (Swayne et al., 2011). Although vaccination is one of the most effective measures for preventing HPAIV, reports on vaccine efficacy in providing protection against HPAIV H5N1 outbreaks have not been encouraging (Suarez and Schultz-Cherry, 2000; Suarez, 2005; Savill et al., 2006).

In developing countries, antiviral drugs for human use, including amantadine hydrochloride and rimantadine, are widely abused for the eradication of AIV in poultry (He et al., 2008; Abdelwhab and Hafez, 2012). Accordingly, antiviral-resistant is likely to be transmitted to human beings from poultry and drug-resistant HPAIV H5N1 epidemics pose a major potential public health threat. In this regard it is worth pointing out that several H5N1 viruses from human and poultry were resistant to amantadine (Cyranoski, 2005; He et al., 2008). Since the late 1990s there has been an increase in recurrent amantadine-resistant HPAIV H5N1 virus strains in poultry in China. This has been attributed to extensive illegal application of the relatively inexpensive amantadine and ribavirin against bird flu in chickens (Parry, 2005; Fan et al., 2015).

*Rhizoma Dryopteris Crassirhizomae* (RDC) has been recorded in Atlas of Chinese traditional Medicine and is rich in phloroglucinol compounds. Generally, RDC has been traditionally used as a herbal medicine for treating various inflammatory and infectious diseases such as tapeworm infestation, colds, and viral diseases in humans. Recent investigations demonstrated that phloroglucinol components extracted from RDC had many pharmacological effects, such as anti-tumor promoting activity (Kapadia et al., 1996), anti-reverse transcriptase activity (Nakane et al., 1991), antioxidant activity (Lee et al., 2003), and antibacterial activity (Lee et al., 2009). Furthermore, flavaspidic acid AB derived from RDC was able to significantly induce IFN-α, IFN-β, and IL1-β expression in porcine alveolar macrophages, suggesting that induction of antiviral cytokines could contribute to inhibition of porcine reproductive and respiratory syndrome virus (PRRSV) replication (Yang et al., 2013a).

Recent evidences showed that RDC contained more than 10 components of phloroglucinol derivatives, such as dryocrassin ABBA, filixic acid ABA, albaspidin AA, albaspidin AP, albaspidin PP, flavaspidic acid AB, and flavaspidic acid PB (Lee et al., 2003), and dryocrassin ABBA accounted for the highest proportion of phloroglucinol components (Gao et al., 2008). In our pilot study, a mixture of RDC and *Fructus mume* was effective in reducing the death rate and viral replication in infectious bursal virus-infected chickens (Ou et al., 2013). The aim of the current study is to evaluate the protective effects of dryocrassin ABBA on HPAIV H5N1 infection in a mouse model.

## Materials and Methods

### Antiviral Drugs

Amantadine hydrochloride was purchased (Sigma-Aldrich, Shanghai, China). Dryocrassin ABBA was isolated from RDC and the purity was determined by high performance liquid chromatography (HPLC). Phenomenex prodigy ODS column (250 mm × 4.6 mm) was employed with a mobile phase of acetonitrile: isopropanol: 0.3% phosphoric acid: 0.1% sodium dodecyl sulfate (50:50:10:5). The sample was detected under 280 nm wavelength at 1.0 mL/min. The final purity was more than 99% of dryocrassin ABBA.

### Virus

H5N1 Avian influenza A/Chicken/Hebei/706/2005 (10^8.7^ELD_50/_0.1ml; H5N1) virus was isolated and identified as previously described. The 50% inhibiting concentration (IC_50_) of amantadine was determined to be 71.6 ± 3.5 μM (He et al., 2008). The virus was donated by Prof. Jian Qiao, China Agricultural University.

### Animal Studies

SPF BALB/C female mice were purchased from Weitong Merial Laboratory Animal Technology Co., Ltd (Beijing, China) and maintained in a negative pressure isolator and provided food and water *ad libitum*. Animal studies were approved by the Animal Care and Use Committee of the China Agricultural University, Beijing, China and conducted in bio-safety P3 lab. In the present study, 120 female BALB/C mice were randomly divided into six groups with 20 mice per group and all mice were maintained in individually ventilated cages (IVC).

The control group was administrated saline intra-nasally while the remaining five groups were inoculated intra-nasally with 10^4.5^ELD_50_ H5N1 viruses in 100 μl saline. On day 2 post inoculation mice received 0.2 ml of dryocrassin ABBA or amantadine hydrochloride by oral gavages. The dosages of the three dryocrassin ABBA groups were 12.5, 18.0, and 33 mg/kg body weight, and 20 mg/kg body weight of amantadine hydrochloride was used as the positive drug control. Mice were given drugs for 7 days from Day 2 to Day 8. Meanwhile, the untreated and control groups received equivalent amounts of physiological saline daily. Body weight, activity, mortality rate, and survival time were monitored daily for 14 days post inoculation.

### Lung Index and Viral Load

Post inoculation on day 7 and on day 14, 5 mice were euthanized to collect the lungs and the lung index was determined as previously described: the dry lung-to-body weight ratio (%) = weight of the whole dry lung/body weight × 100% (Qiao et al., 2009). Afterward, lung tissues were stored at -80°C for further virus quantitation. 50 mg tissue from each mouse was minced to prepare the supernatant from lung homogenates. Lung homogenates were then frozen at -80°C and thawed three times on ice. Homogenates were then centrifuged at 2000 rpm for 10 min at 4°C. Supernatants were transferred to sterile micro-centrifuge tubes and gentamicin added at the concentration of 1000 U/ml for 30 min. Subsequently, 0.1 ml of serial dilutions from 10^0^ to 10^-5^ of the above preparations were injected into the allantoic cavity of 10-days-old embryonated SPF chicken eggs (Weitong Merial Laboratory Animal Co., Ltd., Beijing, China). Post inoculation from 12 to 96 h, any dead or living embryos were collected to obtain allantoic fluid. A hemagglutination assay was used to determine the 50% egg infective dose (EID_50_). Titers of infectious virus were presented as log_10_ (EID_50/_0.1 ml).

### Determination of Cytokines

The cytokine contents in bronchial alveolar lavage fluid (BALF) were used to assess the protective efficacy of drug treatment against AIV H5N1-derived pneumonia. Briefly, three mice were euthanized on day 7 post infection and the lungs were lavaged twice with 1.0 ml sterile saline. Cell suspensions were centrifuged at 1000 rpm for 5 min and the supernatants were collected for cytokines determination. Then the BD CBA Mouse Th1/Th2/Th17 Cytokine Kit (BD, USA) was used to measure the titers of IL-12, IL-6, IL-10, INF-γ, MCP-1 and TNF-α. Lung perfusion fluids were added to tubes with the mixed capture beads and then 50 μL PE of detection reagent were added to all tubes. Samples were incubated, washed and re-suspended for analysis using flow cytometry (BD, USA). Cytokine concentrations were calculated based on standard curve data using FCAP Array software (BD, USA). The results are expressed as mean ± SD. Comparisons between all groups for each cytokine were performed by analysis of variances. Differences between groups were considered significant at *P* < 0.05.

### Statistical Analysis

Data were analyzed with the Statistical Package for Social Science (SPSS, Version 13.0) for Windows and results were expressed as means ± SD. Different group of data were compared by Single factor analysis of variance. Data were considered to be statistically different when *P* < 0.05 or significantly different when *P* < 0.01.

## Results

### Structure and Purity Identification of Dryocrassin ABBA

**FIGURE 1:**
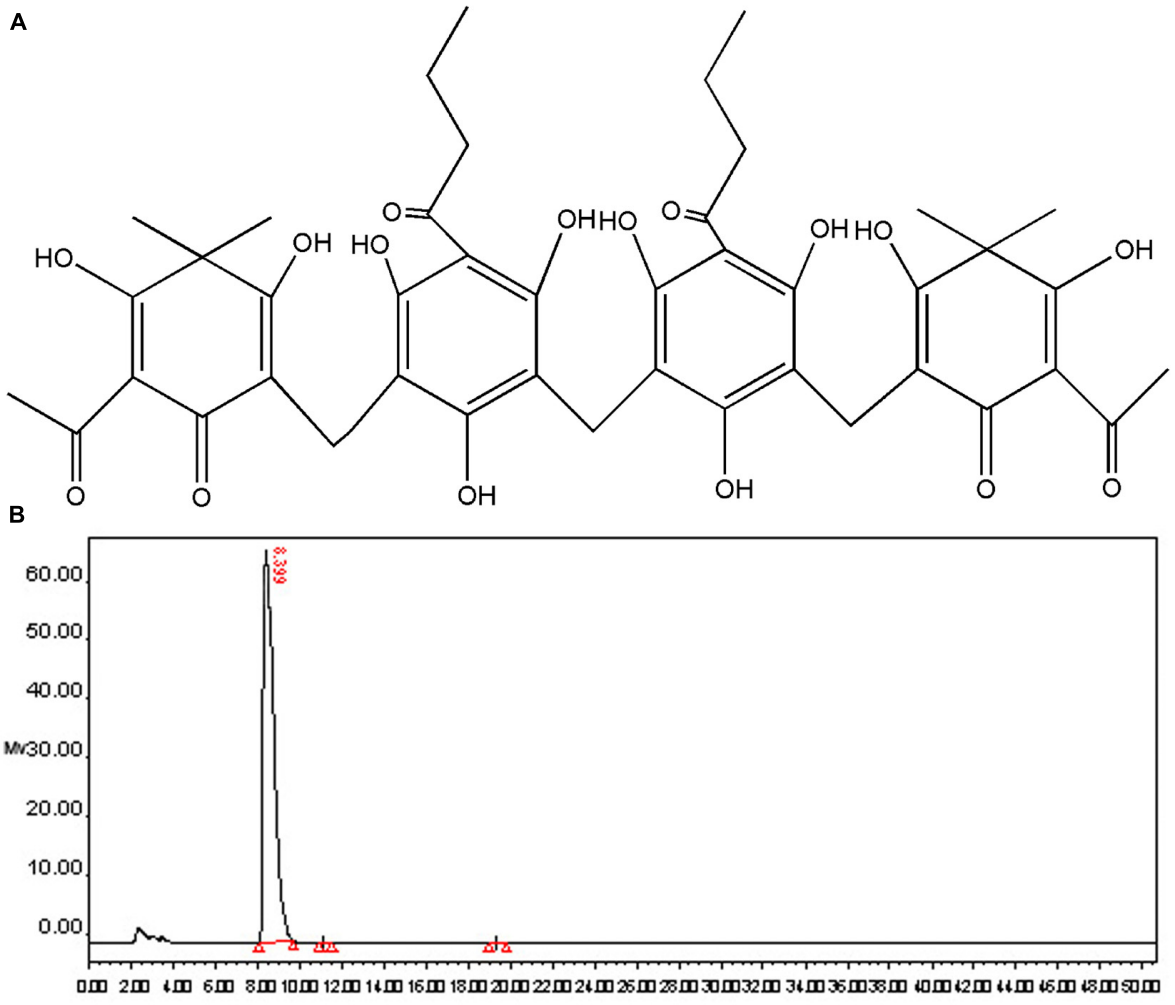
**Chemical structure and purity determination of dryocrassin ABBA. (A)** Chemical structure of dryocrassin ABBA elucidated by related spectroscopy. **(B)** The purity of dryocrassin ABBA was identified as more than 99% by HPLC and the retention time was 8.399 min.

Dryocrassin ABBA (**Figure 1A**), the major phloroglucinol component of RDC, was isolated by column chromatography and re-crystallization and the structure was identified by spectroscopy (data not shown). The purity of dryocrassin ABBA was analyzed by HPLC and the final purity was more than 99% (**Figure 1B**).

### Therapeutic Efficacy of Dryocrassin ABBA against HPAIV H5N1 Infection in Mice

**FIGURE 2:**
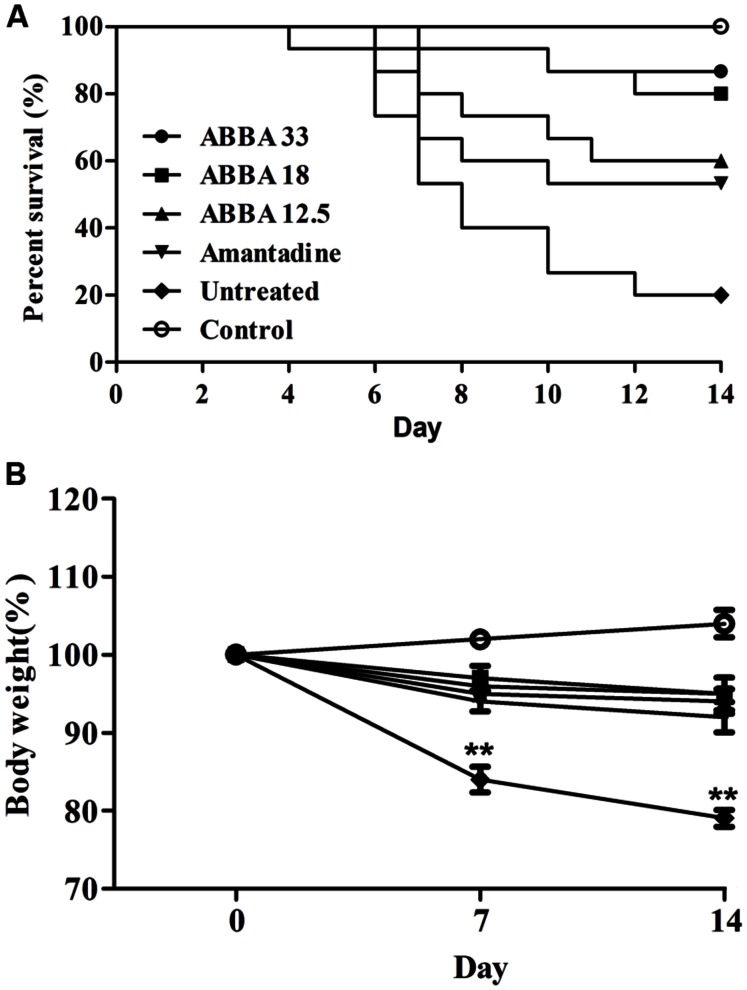
**Therapeutic efficacy of dryocrassin ABBA in mice infected with influenza virus A/Chicken/Hebei/706/2005. (A)** The mice survival rate post administration with 33, 18, 12.5 mg/kg of dryocrassin ABBA or 20 mg/kg of amantadine hydrochloride. The infection control group was inoculated with H5N1 virus without any medication. **(B)** The assessment of mouse body weight post administration with dryocrassin ABBA or amantadine hydrochloride as compared to the untreated or control animals on Day 7 and 14. ^∗∗^ Indicates a statistically significant differences (*P* < 0.01) in body weight when the untreated group was compared with drug-treated groups on day 7 or 14.

Post inoculation with HPAIV H5N1, mice displayed typical signs of infection, such as decreased activity, depression, and poor food intake. On day 2, infected mice began to show weight loss. Animals exhibited abdominal distention and shortness of breath before death, usually on day 5. Dryocrassin ABBA and amantadine significantly reduced mortality, prolonged survival rate, and improved survival time throughout the infection period, compared with the untreated group. Obviously, dryocrassin ABBA exerted a significant dose-dependent antiviral effect in mice. The survival rates were 87, 80, and 60%, respectively in the 33, 18, and 12.5 mg/kg dryocrassin ABBA groups. In contrast, mice administered amantadine hydrochloride showed 53% survival while only 20% mice remained alive in the untreated group (**Figure 2A**). Furthermore, a significant increase in body weight gain was observed in the dryocrassin ABBA-treated groups compared to the amantadine-treated group and the untreated group (**Figure 2B**).

### The Effects of Dryocrassin ABBA on Lung Index and Virus Loads

**FIGURE 3:**
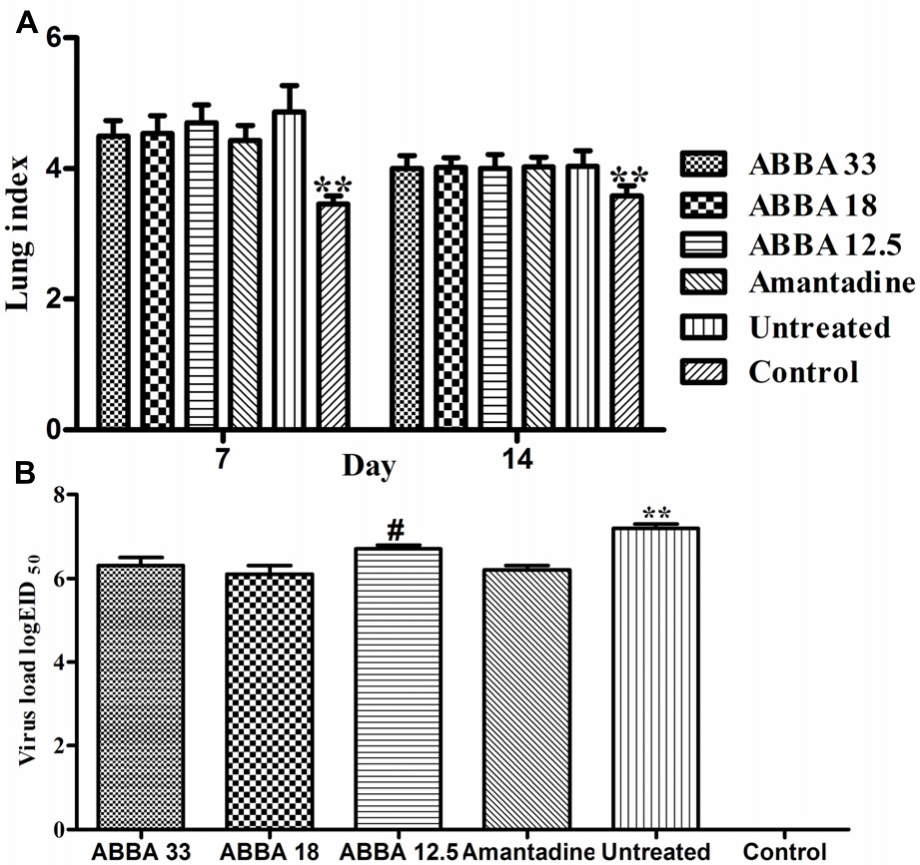
**The effects of dryocrassin ABBA on lung index (A) and virus load (B) post inoculation with avian influenza virus A/Chicken/Hebei/706/2005 (H5N1). (A)** ^∗∗^ Indicates a statistically significant differences (*P* < 0.01) in lung index between the control group and all other virus-infected groups on Day 7 or14. **(B)** ^∗∗^ Indicates a statistically significant differences (P < 0.01) in virus load when the untreated group was compared with drug-treated groups on day 7; #Indicates a statistically significant differences (*P* < 0.05) in virus load when the 12.5 mg/kg dryocrassin ABBA group was compared with amantadine -treated group on day 7.

Post inoculation with H5N1 virus on day 7, mice infected with influenza virus exhibited a significantly greater lung index compared to the control group (*P <*0.01). However, no significant difference was found between the amantadine-treated group and dryocrassin ABBA-treated groups. On day 14, the lung index decreased in virus-infected groups and no statistical significance was found among dryocrassin ABBA-treated groups and the amantadine-treated group (**Figure 3A**). The lung virus loads in both the 33 mg/kg group and 18 mg/kg group were significantly decreased compared to the untreated group *(P <*0.01) and were not significantly different from the amantadine-treated group on day 7 (**Figure 3B**).

### The Effects of Dryocrassin ABBA on BALF Cytokine Levels

**FIGURE 4:**
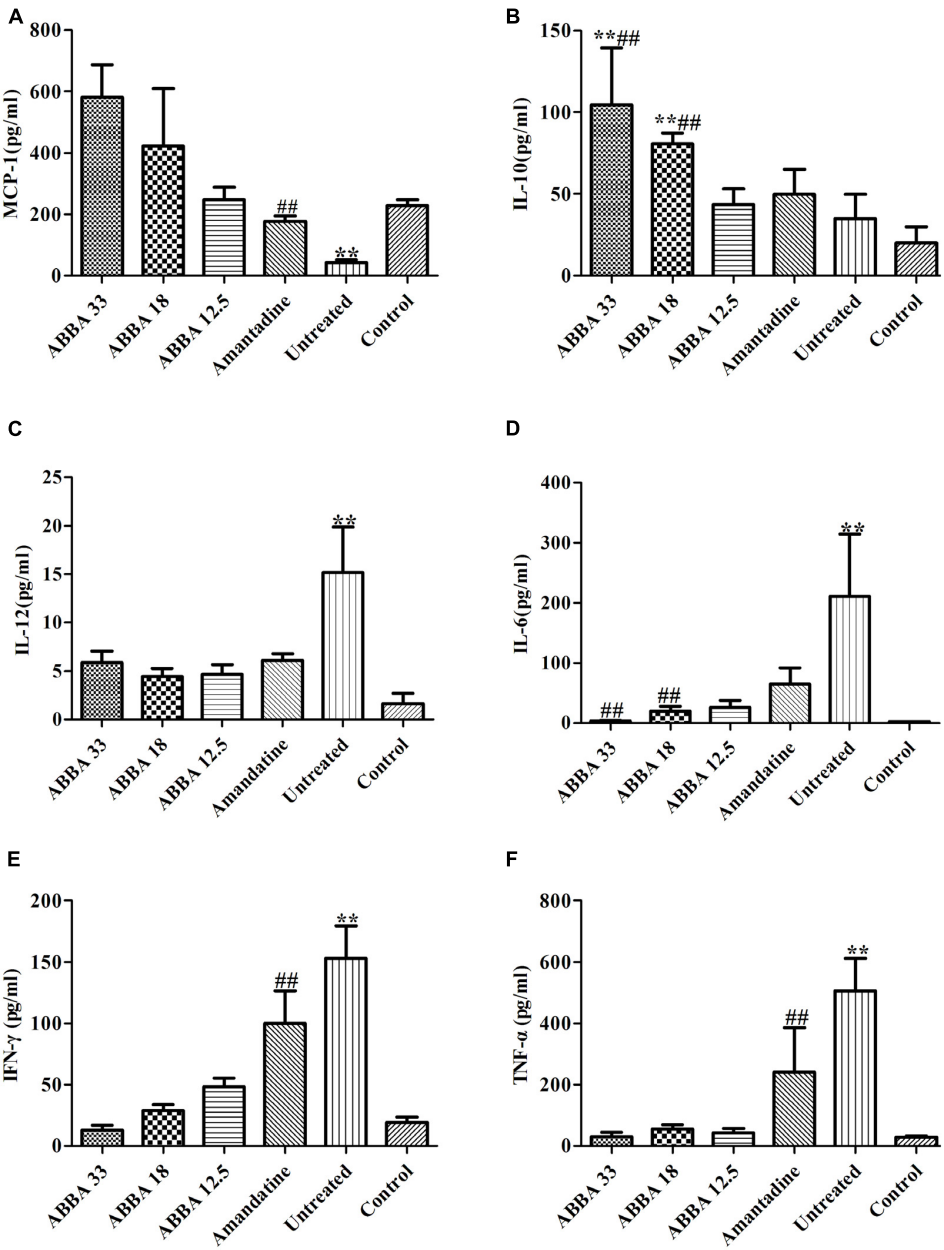
**The effect of dryocrassin ABBA on the levels of the inflammatory cytokines and anti-inflammatory cytokines (TNF-α, IFN-γ, IL-12, IL-6, IL-10, and MCP-1) in BALF by flow cytometry assay on day 7 post infection. (A)** The concentration of MCP-1. ^∗∗^*P*< 0.01 when the untreated group was compared with dryocrassin ABBA-treated groups or amantadine-treated group; ^##^*P*< 0.01 when the amantadine group was compared with dryocrassin ABBA-treated groups. **(B)** The concentration of IL-10. ^∗∗^*P*< 0.01 when the 33 and 18 mg/kg dryocrassin ABBA-treated groups were compared with the untreated group; ^##^*P*< 0.01 when the 33 and 18 mg/kg dryocrassin ABBA-treated groups were compared with the amantadine group. **(C)** The concentration of IL-12. ^∗∗^*P*< 0.01 when the untreated group was compared with the amantadine- or dryocrassin ABBA-treated groups. **(D)** The concentration of IL-6. ^∗∗^*P*< 0.01 when the untreated group was compared with the amantadine- or dryocrassin ABBA-treated groups; ^##^*P*< 0.01 when the 33 and 18 mg/kg dryocrassin ABBA-treated groups were compared with the amantadine group. **(E)** The concentration of IFN-γ. ^∗∗^*P*< 0.01 when the untreated group was compared with the amantadine- or dryocrassin ABBA-treated groups; ^##^*P*< 0.01 when the dryocrassin ABBA-treated groups were compared with the amantadine group. **(F)** The concentration of TNF-α. ^∗∗^*P*< 0.01 when the untreated group was compared with the amantadine- or dryocrassin ABBA-treated groups; ^##^*P*< 0.01 when the dryocrassin ABBA-treated groups were compared with the amantadine group.

MCP-1 and IL-10 increased significantly while IL-12, IL-6, IFN-γ and TNF-α decreased significantly in the 33 and 18 mg/kg dryocrassin ABBA-treated groups compared to the amantadine-treated group and the untreated group on day 7 (*P* < 0.01) (**Figure 4**). The IL-6, IL-10 MCP-1 and IFN-γ displayed dose dependent patterns.

## Discussion

In the present study, mice administered with 33 mg/kg of dryocrassin ABBA showed an 87% survival rate against amantadine-resistant HPAIV H5N1. Moreover, body weight gain increased significantly while lung lesions and virus loads were reduced compared to those of the untreated mice. Moreover, the survival rates of dryocrassin ABBA-treated groups were found to be a dose-dependent manner in the mouse model. Thus dryocrassin ABBA might provide a promising therapeutic approach against bird flu virus in the poultry industry. More importantly, dryocrassin ABBA could be used as a potential novel lead compound which had antiviral effects on amantadine-resistant avian influenza virus H5N1 infection.

Generally, an ideal drug against bird flu should have high efficacy, safety, good quality control, and availability. The threat of a bird flu-derived pandemic requires the development of new therapeutic agents. Amantadine and its derivative rimantadine have been shown to be therapeutically and prophylactically effective against human influenza A viruses (Zlydnikov et al., 1981; Douglas, 1990). In Vietnam, Cambodia, and Thailand, the emergence of amantadine-resistant H5N1 viruses has prompted vaccine development (Cheung et al., 2006). An increasing emergence of adamantine-resistant among seasonal influenza viruses has been reported in isolates of both H1N1 and H3N2 subtypes (Bright et al., 2005, 2006; Dawood et al., 2009). It is urgent to develop an efficient and non-toxic antiviral agent against the outbreak of bird flu.

In the present study, dryocrassin ABBA has been shown to be effective against amantadine-resistant HPAIV H5N1, suggesting a drug alternative against bird flu-resistant strain. As for safety, the maximum toxic dosage (MTD) of dryocrassin ABBA is up to 2000 mg/kg body weight in Sprague-Dawley rats (Hwang et al., 2013) and it is considered a non-toxic substance according to The European Pharmacopeia 7.0 version. From the standpoint of quality control, both (Thin layer chromatography, TLC) and HPLC can be employed to monitor the quality and control the reliability post extraction from traditional Chinese medicine (TCM). Furthermore, RDC grows widely in mountainous areas in the Northeastern China and it is harvested from spring to summer each year, assuring a rich supply of raw material for a large scale manufacture.

Although mice administered dryocrassin ABBA in the present study had a high survival rate and SPF chickens exhibited full protection post inoculation with a virulent infectious bursal disease virus (IBDV), the antiviral mechanism is unclear (Ou et al., 2013). Following administration of dryocrassin ABBA, the lung index of infected mice was significantly reduced compared to that of the amantadine and infection control groups, suggesting the alleviation of lung lesions and inflammation. In the previous report, HPAIV H5N1 virus was used to induce acute respiratory distress symptoms, characterized with by inflammatory cellular infiltration, interstitial and alveolar edema, and hemorrhage (Xu et al., 2006). Our previous data displayed that dryocrassin ABBA could not inhibit the influenza virus replication *in vivo* (data not shown). The discrepancy of anti-influenza activities for dryocrassin ABBA *in vitro* and *in vivo* still needed to be explained. Reduced inflammation would be expected to contribute to an increased survival in infected animals. Dryocrassin ABBA administration might contribute to virus clearance by modifying cytokine balance. The pro-inflammatory cytokines, IL-6, TNF-α, IFN-γ, and IL-12, were significantly reduced while the anti-inflammation factors (IL-10 and MCP-1) increased dramatically in mice administered 33 mg/kg dryocrassin ABBA. Furthermore, IL-10 is a key immuno-regulator during infections, ameliorating the excessive Th1 and CD8+ T cell responses responsible for much of the immunopathology associated with HSV infections (Suvas et al., 2004; Couper et al., 2008). The increased concentrations of IL-10 and MCP-1 in dryocrassin ABBA-treated groups showed a dose-dependent pattern. This dryocrassin ABBA augmentation of MCP-1 and IL-10 might be responsible for a reduced host injury during HPAIV infection. IL-10 is an essential component of this regulatory response in almost all infections. MCP-1 contributed to repair of the alveolar epithelium in influenza pneumonitis (Dessing et al., 2007; Narasaraju et al., 2010). In a recent report, an ethanol extract of *Dryopteris crassirhizoma* displays strong anti-inflammatory activity by suppressing ERK/AP-1 and TBK1/IRF3 pathways, which contribute to its major ethno-pharmacological role as an anti-inflammatory and anti-infectious disease remedy (Yang et al., 2013b).

On other hand, the reduction in pro-inflammatory cytokines (IL-6, IL-12, IFN-γ, and TNF-α) observed in the present study, could have contributed to alleviation of lung lesions. IL-6 and IL-12 have been reported to be key mediators of acute lung injury in mice while IFN-γ and TNF-α were associated with acute lung injury (Kishimoto, 2005; Imai et al., 2008; Hagau et al., 2010). In the current study, the down-regulation of IFN-γ and TNF-α was also dose-dependent in mice administered with dryocrassin ABBA and these cytokines might be involved with influenza virus replication in the lung. IL-10 inhibits MHC class II and co-stimulatory molecule B7-1/B7-2 expression on monocytes and macrophages, and limits the production of pro-inflammatory cytokines (including IL-1α and β, IL-6, IL-12, IL-18, and TNF-α) and chemokines (MCP1, MCP5, RANTES, IL-8, IP-10, and MIP-2; Couper et al., 2008).

Finally, dryocrassin ABBA might also be associated with virus clearance by activation of signal transduction pathways. Aspidin PB was capable of inducing apoptosis, as measured by modulating the PI3K/Akt/GSK3b pathway (Sun et al., 2013). Antiplatelet and anti-inflammatory effects of phloroglucinol are related to inhibition of COX, ROS, and TXA2 production as well as ERK/p38 phosphorylation in platelets (Chang et al., 2012).

## Conclusion

In mice infected with an amantadine-resistant H5N1 strain, dryocrassin ABBA provided a significant improvement in survival by alleviation of lung lesions and virus loads, effects was probably mediated by decreased pro-inflammatory cytokines and increased anti-inflammatory cytokines. Dryocrassin ABBA warrants further study as a promising therapeutic agent against HPAIV H5N1. Future investigations will be performed to understand the mechanisms and expand the potential application of dyocrassin ABBA against other viral diseases.

## Conflict of Interest Statement

The authors declare that the research was conducted in the absence of any commercial or financial relationships that could be construed as a potential conflict of interest.
