# Corrigendum: Dryocrassin ABBA, a novel active substance for use against amantadine-resistant H5N1 avian influenza virus

**DOI:** 10.3389/fmicb.2016.01464

**Published:** 2016-09-15

**Authors:** Changbo Ou, Qiang Zhang, Juan Wang, Guojiang Wu, Ningning Shi, Cheng He, Zengping Gao

**Affiliations:** ^1^College of Animal Science, Henan Institute of Science and TechnologyXinxiang, China; ^2^Key Lab of Animal Epidemiology and Zoonosis, Ministry of Agriculture, College of Veterinary Medicine, China Agricultural UniversityBeijing, China; ^3^School of Chinese Materia Medica, Beijing University of Chinese MedicineBeijing, China; ^4^College of Life Science, Agricultural University of HebeiBaoding, China; ^5^College of Pharmacy, Hebei Medical UniversityShijiazhuang, China

**Keywords:** dryocrassin ABBA, influenza virus H5N1, phloroglucinol compound, antiviral activity, Rhizoma Dryopteridis Crassirhizomatis, amantadine

In the original research article, Juan Wang and Zengping Gao were not included in the author list. They have now been included along with the affiliation: School of Chinese Materia Medica, Beijing University of Chinese Medicine, Beijing, China.

In addition, Zengping Gao has been included as a corresponding author.

Finally, the authors unintentionally did not acknowledge funding from the National Natural Science Foundation of China No.31270400.

The authors apologize for these oversights. These errors do not change the scientific conclusions of the article in any way.

The original article has been updated.

## Conflict of interest statement

The authors declare that the research was conducted in the absence of any commercial or financial relationships that could be construed as a potential conflict of interest.

